# Sleep Quality Daytime Nap and Dreaming in Later Life Associations with Biopsychosocial Characteristics

**DOI:** 10.1192/j.eurpsy.2026.11796

**Published:** 2026-07-31

**Authors:** A. Nakou, C. Tsironis, E. Dragioti, E. Bartzou, N. Zagorianakou, S. Mantzoukas, M. Gouva

**Affiliations:** University of Ioannina, Ioannina, Greece

## Abstract

**Introduction:**

Sleep quality in older adults is a critical indicator of well-being and is influenced by biological, psychological, and social factors. Daytime napping and dreaming represent additional dimensions of sleep that may reflect physical health, psychological functioning, and social relationships.

**Objectives:**

The aim of this study was to investigate the relationship between biopsychosocial characteristics of older adults and sleep quality, daytime napping, and dreaming.

**Methods:**

A total of 608 older adults (aged 65-99 years) participated in the study. Participants completed a questionnaire including demographic and social characteristics (sex, marital status, educational level, number of children), measures of physical and mental health (SF-36 subscales: MH, SF, GH, MENTAL HEALTH), and sleep-related variables (“Good Sleep,” “Daytime Nap,” “Dreams”). Descriptive statistics, Pearson’s correlations, and group mean comparisons were conducted.

**Results:**

Sleep quality was positively correlated with indicators of mental health: *MH* (r=0.30), *SF* (r=0.29), *MENTAL HEALTH* (r=0.25), and *GH* (r=0.24, p<0.001). Daytime napping differed by sex (r=-0.30) and by marital status (means 2.8–3.7). Having children was associated with better sleep quality (M=5.0 vs. 3.6) and more intense dream experiences (M=3.1 vs. 1.0). The number of children was related to longer daytime napping (M=4.3 vs. 3.1) and higher sleep quality (M=4.7 vs. 3.8).

**Conclusions:**

The findings demonstrate that sleep quality in older adults is shaped by both psychological factors (mental well-being) and social parameters (family structure, children). Daytime napping and dreaming reflect the interaction between biological and social characteristics, underscoring the importance of a biopsychosocial approach in understanding and improving sleep in late life.

**Disclosure of Interest:**

None Declared

